# Social genomics and cancer – untangling nature from nurture in squamous cell carcinoma outcomes

**DOI:** 10.3389/fonc.2026.1661519

**Published:** 2026-07-20

**Authors:** Parnian Kheirkhah Rahimabad, Arash Shaban-Nejad, Robert Davis, D. Neil Hayes, Liza Makowski, David L. Schwartz

**Affiliations:** 1 Departments of Radiation Oncology, College of Medicine, University of Tennessee Health Science Center, Memphis, TN, United States; 2 Department of Pediatrics, Center for Biomedical Informatics, College of Medicine, University of Tennessee Health Science Center, Memphis TN, United States; 3 Division of Hematology-Oncology, Department of Medicine, College of Medicine, University of Tennessee Health Science Center, Memphis, TN, United States; 4 Departments of Preventive Medicine, College of Medicine, University of Tennessee Health Science Center, Memphis, TN, United States

**Keywords:** cancer outcomes, epigenetics, social determinants of health, social genomics, squamous cell carcinoma

## Abstract

**Background:**

Outcomes in squamous cell carcinomas (SCCa) of the head and neck, esophagus, and lungs are increasingly linked to the complex interplay between social determinants of health (SDoH) and biological pathways. The emerging field of social genomics provides mechanistic insight into how the environmental and socioeconomic conditions may influence tumor biology through stress-mediated pathways, epigenetic modifications, and altered gene expression.

**Objective:**

This review explores the role of adverse socioeconomic conditions such as neighborhood deprivation in shaping SCCa outcomes and the potential underlying mechanisms. In response to chronic stress, hypothalamic-pituitary-adrenal (HPA) axis and sympathetic nervous system become activated, leading to dysregulated immune signaling and proinflammatory gene expression pattern collectively known as the Conserved Transcriptional Response to Adversity (CTRA). We discuss epigenetic modifications including DNA methylation (DNAm), histone modification, and micro RNA (miRNA) dysregulation as potential mediators of these stress-related effects. Studies show that SCCa may have distinct race- and neighborhood-specific DNAm patterns including differential methylation of *PAX5*, *HOXA7*, and *TFPI* genes, and altered expression of xenobiotic metabolism genes regulated by Nrf2, a major stress response transcription factor. Therapeutic strategies targeting these biological mediators including β-adrenergic blockers, DNA methyltransferase inhibitors (e.g., azacytidine, decitabine), histone deacetylase inhibitors (e.g., vorinostat), and BET inhibitors have shown variable efficacy in preclinical and clinical SCCa models. Incorporating social context into tumor genomic analysis through geospatial modeling and neighborhood epigenomic profiling may offer a novel opportunity for identifying population-level cancer risk patterns and therapeutic targets.

**Conclusion:**

Social genomics provides a deeper understanding of the interaction of socio-environmental exposures with the epigenome and tumor biology influencing disparities in SCCa outcomes. Future research should integrate geospatial and multi-omics data to inform personalized cancer prevention and treatment strategies.

## Introduction

*“Geography is destiny.”*

― Napoleon Bonaparte, among countless others

Location and fate are deeply intertwined. Local abundance of material and social resources is imprinted upon human traits, influencing future prosperity or peril. Neighborhoods serve as social ecosystems, with innumerable features impacting health. A person’s immediate environment influences an array of opportunities for healthy behavior, such as access to fresh produce, green spaces for exercise, or public transportation options to reach healthcare providers. Neighborhood deprivation has been used as a proxy for the complex web of social health determinants of health (SDoH) (1, 2). Neighborhood-level disparities reduce access to cancer screening (3, 4), treatment (5, 6), and enrollment in clinical trials (7). Cancer incidence, treatment quality, and survival rates are demonstrably worse in socioeconomically distressed populations across the United States and abroad (8–13).

As an example, a recent cohort study of 5027 women with breast cancer showed that those from disadvantaged neighborhoods had shorter breast cancer-specific survival compared to those from advantaged neighborhoods, even after accounting for various individual-level factors such as demographics, comorbidities, risk factors, and tumor characteristics (14). The emerging concept of “social genomics” suggests that, in addition to shaping access to resources, social adversity in disadvantaged neighborhoods influences survival outcomes through discrete biological mechanisms at a molecular level (15–17).

Outcome disparities are particularly evident for Squamous Cell Carcinomas (SCCa) of the head and neck (18), esophagus (19), and lung (20). Squamous epithelial cells lining the oral cavity, pharynx, larynx, esophageal mucosa, and bronchial airways are uniquely positioned at the interface between the organism and its external environment and are highly influenced by inhaled and ingested exposures. The major carcinogens driving SCCa at these sites, including tobacco smoke, alcohol, and occupational air pollutants, follow steep socioeconomic gradients (21, 22). SCCa of different sites share recurrent genomic profiles and overlapping patterns in gene expression and epigenetic modification (23). Therefore, SCCa provides a biologically, clinically, and socially relevant model to study SDoH and cancer outcome disparities.

Candidate pathways by which social stressors impact health status include signal transduction, inflammation, and epigenetic modifications (17, 24). Social hardship can induce a chronically stressed physiologic state, which in turn activates the sympathetic nervous system and the hypothalamus-pituitary-adrenal (HPA) axis, releasing neuroeffector substances like cortisol and norepinephrine (25). Under normal conditions, cortisol activation of the glucocorticoid receptor via (HPA) axis induces the expression of anti-inflammatory genes and simultaneously suppresses NF-κB proinflammatory transcription factors. However, chronic stress blunts these regulatory mechanisms leading to a proinflammatory state in the transcriptomic profile of leukocytes (24). The sympathetic nervous system exerts its downstream effects via beta adrenergic receptors, particularly on immune cells, leading to blocking anti-tumor immune response (26). Activation of beta receptors induces macrophage recruitment promoting metastasis (27). Additionally, norepinephrine released by local tumor nerve endings induces angiogenesis in a beta 2-dependent process (25, 28). This stress-induced genetic regulation results in gene expression patterns collectively known as the Conserved Transcriptional Response to Adversity (CTRA). The CTRA response is characterized by high levels of proinflammatory gene expression and low expression levels of genes involved in innate immunity (29).

Unlike direct mutational changes to the genome, an individual’s epigenome is readily altered through histone modifications, noncoding micro RNAs (miRNA), chromatin remodeling, and DNA methylation (DNAm). These modifications can be prompted by external stressors, followed by downstream impact on gene expression and phenotype. Human and animal studies have shown differential DNAm profiles in response to psychosocial stress (24). Socioeconomic status (SES) indices and adverse social exposures have been associated with altered DNAm levels of genes involved in inflammation, suggesting mechanisms by which environmental experiences get embedded into cells (30, 31). Accelerated epigenetic aging, a state where one’s biological age (assessed by their DNAm levels) exceeds chronological age (32), has been implicated in several chronic diseases, including cancer (33). Social hardship leads to accelerated epigenetic aging, particularly in children (34–36). Histone modification, including acetylation and methylation, is another mechanism by which stress can alter gene transcription (37). In murine studies, differential histone modification has been implicated in distinct susceptibility in response to chronic stress (38, 39). Small non-coding RNAs, including miRNAs, are highly conserved molecules with a critical role in the regulation of gene expression (40). By regulating stress signaling pathways, miRNA modifies the response to stress (41). Tumor cells display dysregulated miRNA expression patterns compared to normal tissue; these tumor-specific signatures show sufficient discriminatory power to classify cancer type and distinguish malignant from benign tissue (41).

## Social genomics and cancer disparities

Social genomics provides mechanistic perspectives to understand and potentially reduce health disparities through biologically informed interventions to mitigate patient-level impact from adverse population-level conditions. Neighborhood characteristics, SES and race/ethnicity, among other SDoH, have been shown to influence cancer outcomes (42–44). Early studies on common cancer diagnoses, particularly breast and prostate cancer, suggest that race/ethnicity-specific biological risk factors contribute to population-level cancer disparities. Breast cancer remains the second leading cause of cancer mortality in American women (45). Although Black and White women have similar rates of breast cancer diagnosis, Black women experience a higher incidence of diagnosis at a younger age (46) and are more likely to present with more aggressive forms, such as triple negative breast cancer (47). In a large Georgia cohort of 36,795 patients, neighborhood deprivation was associated with increased breast cancer mortality among White women; however, this association was not observed among Black women (48). This suggests that factors beyond neighborhood deprivation, including biological mechanisms not captured by socioeconomic indices, may drive the excess breast cancer mortality observed in Black women. Several race-associated biological risk factors have been suggested to explain disparate risk in Black and White women. For example, *SOS1*, a regulator of Ras signaling, is overexpressed in Black women. Likewise, cancer disparity-linked genes *CRYBB2* and *CRYBB2P1* are overexpressed in breast tumors of Black women compared to White women, promoting tumor growth (49). In a recent cohort study, obesity and high serum leptin levels were associated with an enhanced risk of early onset triple negative breast cancer in Black women (45).

Residence in persistently impoverished neighborhoods has been associated with aggressive breast cancer phenotypes (50) and inferior survival outcomes (43). Women living in disadvantaged neighborhoods experienced worse survival rates, even after accounting for personal factors, tumor characteristics, and treatment (14). Similarly, advanced geographical modeling of the Surveillance, Epidemiology, and End Results (SEER) cancer registry data has confirmed co-localization of cancer risk exposures, obesity rates, and breast cancer mortality at the U.S. county level, underscoring the influence of SDoH on cancer outcomes (51). Recent work has begun to explore interactions between social and biological breast cancer risk factors. One study confirmed race-specific patterns of breast tumor DNAm and gene expression, highlighting biological phenotypes specific to Black women (52). Another study prospectively linked neighborhood deprivation to high-risk tumor transcription factor expression and activation of neurologic stress responses (53). The pro-inflammatory CTRA gene expression profile has been linked to ER negative breast cancer which disproportionately affects Black women (54).Neighborhood deprivation has been shown to upregulate the CTRA gene expression in breast cancer patients’ leukocytes, with higher expression correlating with more aggressive tumor characteristics and worse overall survival, directly linking social adversity to a stress-related biological pathway driving cancer outcome disparities (55).

Prostate cancer is the leading cancer diagnosis in U.S. men. Both the incidence and mortality rates of prostate cancer are higher in Black patients (56). Despite their elevated risk, Black men are susceptible to being screened less frequently than White men and to present with more aggressive disease (57). Emerging evidence suggests that SDoH, particularly neighborhood disadvantage, is associated with both African American race (58) and advanced stage at the time of diagnosis (59). This link suggests that chronic stress, referred to as allostatic load, interacts with host physiology (epi/genomic expression, neuroendocrine responses, and inflammation) to influence prostate cancer risk (60, 61). Molecular studies support this intersection of social and biological factors. Neighborhood disadvantage has been significantly linked to the expression of inflammation-related genes in serum (62). Further, a cross-sectional study found that men from disadvantaged neighborhoods have higher expression level of several stress-related genes in prostate tumors (56). This study was one of the first to directly associate neighborhood disadvantage with prostate tumor RNA expression, highlighting a potential biological mechanism through which social factors contribute to cancer disparities. Beyond static measures of neighborhood disadvantage, dynamic neighborhood change may also shape cancer outcomes. Neighborhood gentrification, i.e., the process by which low-income areas undergo economic reinvestment and demographic restructuring, has been associated with prostate cancer in both African American and White men, with heterogeneous effects across subgroups (63). These findings suggest that changing neighborhood socioeconomic conditions may influence prostate cancer outcomes, adding a temporal dimension to the social genomics of cancer disparities complementing cross-sectional measures of neighborhood deprivation.

Our group has investigated neighborhood-level risk factors of obesity among adults in Shelby County in Southwestern Tennessee via a geospatial machine learning approach. This has allowed us to examine how obesogenic neighborhood environments influence prostate cancer risk and mortality in the large and geographically dispersed Southern Community Cohort Study (64). We found that living in a socioeconomically disadvantaged neighborhood correlated with higher prostate cancer risk, particularly among Black men. Additionally, restaurant and retail food environment indices, as key markers of an obesogenic environment, were associated with elevated prostate cancer risk. This association was more pronounced in overweight White populations. Furthermore, residing in a low-SES neighborhood and/or areas with the least walkable spaces was associated with a higher risk of prostate cancer mortality. Our findings align closely with previously observed associations linking minoritized neighborhood location, race, and environmental pollution levels to cancer risk in Western Tennessee (65). Notably, Jia et al. found that census tracts with higher proportions of African American residents in Memphis, Tennessee, had significantly elevated estimated cancer risk from air toxics (including benzene, chloroform, and formaldehyde) with cumulative cancer risk showing a strong positive correlation with racial composition (65). This further emphasizes the tight geographic overlap of socially defined environmental burdens which can influence individual-level cancer risk. The molecular and epigenetic mechanisms underlying these neighborhood-associated disparities are discussed in depth in the following section.

## An epigenetic link between socioecological hardship and cancer risk

Neighborhood-level adversity and social hardship influence cancer outcomes not only through access barriers, but by leaving a distinguishable molecular imprint on tumor biology. In this section, we discuss the biological mechanisms linking social adversity exposures to cancer risk, with emphasis on epigenetic modification as a key player. Characterization of epigenetic variations across individuals and populations offers an opportunity to understand how social and environmental factors influence cancer outcomes (31). Studies have identified a distinct race-specific DNAm expression patterns across breast, prostate, colorectal, lung, and head and neck cancers (66, 67). For example, colorectal cancers in Black patients have a different DNAm pattern, with 1,588 hypermethylated and 100 hypomethylated differentially methylated regions (DMR), compared to cancers in White patients, which show 109 hypermethylated and 4 hypomethylated DMRs (68). Racial differences exist not only across baseline DNAm levels linked to cancer genes but also methylation responses to the environment. Park et al. showed that the effect of smoking dose on DNAm of certain genes was distinct in Native Hawaiian smokers relative to White or Japanese American peers (69). This could explain why Hawaiian smokers face a higher risk of lung cancer than White smokers, even when exposed to the same levels of smoking.

The importance of neighborhood characteristics in social genomics may help elucidate geographical disparities in cancer outcomes and the potential role of epigenetics. Factors such as neighborhood SES, perceived neighborhood stress, physical activity opportunities, availability of healthy food options, and proximity to healthcare services have been shown to influence cancer survivorship (70). These neighborhood-level exposures may be reflected in an individual’s epigenome (71). In a U.S. urban setting, reduced neighborhood greenness was linked to accelerated epigenetic aging, particularly pronounced among Black individuals (72). Miller-Kleinhenz et al. showed that neighborhood-level adversities such as contemporary redlining is associated with distinct epigenetic patterns in breast cancer, including differential DNAm of genes implicated in inflammation, immune function, and stress response (73). Neighborhood-level differences in cancer outcomes can also be observed across urban and rural settings, with rural populations experiencing higher morbidity and mortality (74). While most research on environmental risk factors has focused on urban areas, rural populations often face substantial exposure to serious environmental pollutants from agriculture, mining, and industrial operations (75). Compounding these exposures, rural populations in high-poverty areas have higher measures of SDoH burden, less access to healthcare, and lower health literacy (74). Conversely, urbanicity may be associated with high-risk health behaviors such as reduced physical activity and greater consumption of ultra-processed foods (76). Rural residence itself has been associated with a higher incidence of cancer, particularly in the Southeastern U.S. (77). A retrospective cohort study using the National Cancer Database (2004–2015) found that head and neck cancer patients from rural areas had worse survival outcomes than patients from urban areas. This urban-rural disparity was most pronounced in Black patient populations relative to White (78). Recent studies suggest that underlying epigenetic differences between rural and urban residents may be potential mechanistic factor driving observed differences in health outcomes (79).

Early life and childhood are critical periods marked by a heightened sensitivity to social and environmental exposures. Social hardships experienced during childhood can get biologically “embedded” within individuals, giving rise to disparities in health outcomes later in life (80). Our group performed a scoping review to catalog emerging connections between adverse childhood experiences (ACE), cancer incidence, and cancer-specific mortality (81). A recent meta-analysis of 18 studies (n=406,210) found that individuals with at least two ACEs were at an increased risk for cancer (82) while findings from another review further associated a history of physical and psychological abuse with increased cancer risk in adulthood (83). Although the potential mechanisms underlying the association of ACE and health disparities are not yet clarified, there are likely roles present for epigenetics, biological aging, and chronic inflammation (84, 85). Social hardship (e.g. low SES and high psychological stress) and biological aging (e.g. telomere length and genomic instability) have been associated with increased risk for a range of chronic diseases, including cardiovascular disease, metabolic disorders, and cancer (84, 86). Leveraging three decades of data from the National Heart, Lung, and Blood Institute (NHLBI) Growth and Health Study (NGHS), investigators prospectively confirmed that low childhood SES and perceived childhood stress are independently and additively associated with two established biological markers of mortality risk: insulin resistance and epigenetic aging (87).

The mechanistic effects of ACEs on epigenetic changes related to cancer risk have not yet been fully investigated, but there are clues to follow. Adverse social exposures in early life have been linked to altered inflammation levels later in life, potentially through impacting DNAm patterns (31, 88). These alterations in the DNAm due to adverse social conditions in early life and the downstream proinflammatory phenotype may result in an individual’s increased risk of cancer development. A study of adolescent girls (n=44) with a history of multiple ACEs showed methylation changes in genes related to cancer processes before and after a one-week intensive group program (89). There are also available data suggesting that effects of ACEs on cancer risk could be mediated through inflammatory pathways. One study surveyed 408 adults with hepatobiliary-pancreatic cancer using the Traumatic Events Survey; the authors uncovered a link between lower levels of interleukin 2 (IL-2) and pre-existing exposure history to ACEs, particularly those associated with upheaval between parents (90). Another review of nine articles, including 2931 participants, found an association between certain ACEs and increased plasma levels of interleukin 6 (IL-6), C-reactive protein (CRP), soluble tumor necrosis factor receptor type II (sTNF-RII), interleukin 1 receptor antagonist (IL-1ra), and subsequent increased risk of developing breast cancer (91).

Trauma-related epigenetic modifications may unfortunately even be heritable (92, 93). Stress can impact gamete epigenetics in preclinical models (94). There is evidence that epigenetic inheritance can be transgenerational. Trauma experienced by a grandmother while pregnant can alter DNAm patterns in grandchildren (95). There remains a significant gap in our understanding of potential roles of epigenetics in the transmission of stress response and secondary cancer risk across generations, but the opportunity to disrupt intergenerational links in cancer burden would be a unique opportunity to deliver preemptive health benefits to children not yet even born.

## Social genomics and disparities in squamous cell carcinomas outcomes

Turning attention to SCCa of the upper aerodigestive tract, what is currently known? Lung (LSCCa), head and neck (HNSCCa), and esophageal squamous cell carcinomas (ESCCa) are closely associated with a specific behavioral risk which has become increasingly associated with lower SES: tobacco exposure (96–99). Socially vulnerable populations smoke more often and face a higher incidence of these cancers. Populations living in segregated, high vulnerability neighborhoods smoke more frequently, receive less aggressive treatment and suffer higher mortality from non-small cell lung cancer (NSCLC) (100). Furthermore, neighborhood-level deprivation has been shown to affect tumor DNAm pattern and survival outcomes in NSCLC. Particularly, DNAm at certain genes, including *HOXA7*, has been associated with disease stage and correlated with indices of neighborhood deprivation (1).

In HNSCCa, social vulnerability and community deprivation are predictive for advanced presentation (101) and low-quality treatment (e.g. clinically significant delays in postoperative radiation treatment) (102). Guerrero-Preston et al. (67) demonstrated differences in DNAm levels in the promoter of the genes previously associated with HNSCCa between Black and White patients. The authors further analyzed DNAm levels in the *PAX5* gene promoter across two distinct zip codes. Adjusted for race, patients from areas with higher annual income and lower home vacancy rates had lower DNAm levels and better survival outcomes than those from the disadvantaged zip code. Black patients with *PAX5* methylation had poorer survival compared to Whites, after adjusting for TNM stage, education level, and zip code. This suggests that social and racial factors impact HNSCCa at a molecular and epigenetic level.

Significant differences exist in the epigenetic profiles of cancers among different racial groups. However, it remains unclear how individual vs. population-level exposure and host characteristics contribute to such variation. Esophageal cancer histologies have marked racial predilections; adenocarcinoma predominates in Whites while ESCCa presents more frequently in Blacks. ESCCa in Black individuals has poor survival outcomes (103) and a distinct epigenetic landscape. Erkizan et al. investigated the gene expression profile in Black ESCCa patients (104). Transcriptome analysis showed aberrant expression in nuclear factor erythroid 2-related factor 2 (Nrf2)-mediated stress response and xenobiotics metabolism. Nrf2 modifies expression of antioxidant enzymes in response to oxidative stress (105). Several genes regulated by Nrf2 encode critical enzymes involved in the metabolism of alcohol, a known risk factor for ESCCa (104). Another study comparing White and Asian patients with ESCCa found differences in DNAm levels of the tissue factor pathway inhibitor (*TFPI*) gene. Comparing tumor transcriptomes from Asian and White patients revealed 63 differentially expressed genes. These genes were linked to biological pathways such as cell adhesion potentially involved in the tumor microenvironment and progression (106). In a large cohort study across multiple cancer types, including HNSCCa, Lee et al. found genetic ancestry to be significantly associated with overall survival disparities in a subset of cancers, including HNSCCa. They showed that ancestry-associated differences in gene regulation, i.e., differential gene expression accompanied by differential DNAm patterns, had a greater contribution to survival disparities than population-specific genetic mutations (107). These findings suggest that environmental exposures may become biologically embedded through epigenetic mechanisms and contribute to cancer outcome disparities across populations (108).

Several limitations in studies on SDoH and epigenetics of SCCa are worth noting. First, causal relationships between SDoH and epigenetics of SCCa cannot be established solely from the limited existing literature, which is composed of observational association studies. In addition, SDoH are highly interdependent and mutually reinforcing, creating challenges in disentangling temporality, mediation, causal ordering and concerns for residual and structural confounding. For example, tobacco use which is commonly used as an individual-level covariate follows a strong SES gradient shaped by education, income, and neighborhood context. Adjusting for smoking may partially control for the social exposures of interest, complicating interpretation of independent SDoH effects.

Future longitudinal studies integrating detailed exposome data with multi-omics profiling and clinical outcomes are needed to clarify temporal relationships and investigate causal relationships. Interventional studies targeting modifiable epigenetic pathways may help determine whether epigenetic changes represent mediators of social adversity or merely reflect broader structural disparities.

## Could epigenetic data lead to socially-informed treatment of SCCa?

There is a need for mechanistic insights to reveal potential targets for therapeutic intervention of environmentally instigated cancer risk. A useful example of a druggable target would be stress-induced overstimulation of the sympathetic nervous system. Inhibition of the sympathetic nervous system by beta blockers unlinks chronic stress from downstream pro-inflammatory gene expression patterns in adults (109). Propranolol administration delayed fibrosarcoma progression in mice (109, 110). In murine models of colon and breast cancers, Chen et al. showed that blockade of beta 2 receptors increases the efficacy of radiation therapy (111). Treating oral squamous cell carcinoma (OSCCa) *in vitro* with propranolol decreased tumor viability and sensitized cells to cisplatin and 5-fluorouracil (112). Another murine model of recurrent/metastatic HPV+ HNSCCa demonstrated that propranolol suppresses tumor progression and metastasis *in vivo* and shows additive benefits combined with the standard chemoradiation therapy (113). However, in two follow-up studies on HNSCCa patients, beta blocker usage was associated with poorer survival outcomes (114, 115). These conflicting results regarding the use of beta blockers in HNSCCa have been attributed to the complex downstream effects of different beta adrenergic receptor activations on cancer mechanisms.

Agents modifying DNAm have risen as novel therapeutic agents for cancer treatment. Azacytidine, a DNA methyltransferase (DNMT) inhibitor has received FDA approval for certain clinical indications in the treatment of certain hematologic malignancies. Usage of azacytidine for the treatment of solid tumors is under investigation. Mouse models have shown that azacytidine inhibits HPV+ HNSCCa growth and invasion (116). An ongoing phase 2 clinical trial is investigating addition of azacytidine to nivolumab in treatment of HPV+ oropharyngeal cancer (117). Decitabine is another DNMT inhibitor that is used in myelodysplastic syndrome treatment. Pre-treatment of cisplatin-resistant HNSCCa cell lines with decitabine shifted DNAm and gene expression towards a cisplatin-sensitive phenotype (118). Hypermethylation of promoters of tumor suppressor genes have been observed in NSCLC. DNAm modifiers have been shown to reverse hypermethylation, hence, providing a potential therapeutic opportunity for NSCLC. Treatment of cell lines of LSCCa with azacytidine reversed hypermethylation of a tumor suppressor (*RASSF1a*) promoter and inhibited growth (119).

Chronic stress can also alter histone modification (120, 121). Targeted modifiers of histone acetylation may alternatively offer promise in selected cases of SCCa. Apcidin inhibits OSCCa proliferation *in vitro* and *in vivo* murine models (122), while trichostatin A, suppresses the growth of ESCCa cell lines (123). Another *in vitro* study showed increased expression of apoptotic genes and decreased expression of genes related to tumor invasion in tongue SCCa cell lines treated with azacytidine and Trichostatin A (124). Vorinostat, a histone deacetylase inhibitor (HDAC), was added to cisplatin/radiation therapy in advanced stage HNSCCa patients in a single-arm study (125). This combination regimen was well-tolerated and demonstrated tumoricidal activity. In a partially randomized clinical trial, usage of vorinostat in combination with capecitabine in treatment of recurrent and/or metastatic HNSCCa showed limited activity (126) Another HDAC, namely FK228, showed growth inhibition of ESCCa in an *in vitro* and *in vivo* murine study (127).

By binding to histones and other nuclear proteins, Bromodomain and extra-terminal (BET) proteins promote gene expression (128). BET proteins have been extensively implicated in proinflammatory and immunomodulatory processes leading to carcinogenesis (128, 129). In preclinical studies, BET inhibitors (BETi) have shown promise in treatment of different cancers when combined with other agents such as immune checkpoint inhibitors (ovarian cancer, NSCLC) and other epigenetic modifying agents such as HDACs (myeloid leukemia, breast cancer) (130). BRD4, a key BET protein, is a transcription suppressor and inhibits the expression of MHC I molecules. BRD4 is implicated in HNSCCa where its increased expression leads to decreased infiltration of CD8+ T cells (131). In murine models of HNSCCa, BET inhibition resulted in heightened sensitivity to treatment with anti-PD1 checkpoint inhibitors and increased antitumor immunity by CD8+ T cells (131). In another *in vitro* study, the BRD4 inhibitor halted progression through multiple mechanisms. It suppressed ESCCa proliferation while increasing ESCCa migration via autophagy promotion (132).

It remains unclear to what extent the epigenetic aberration in SCCa can be attributed to social adversities and how much of it can be alleviated by epigenetic modifiers. Population-level epidemiologic studies will be necessary to corroborate associations and mechanistic links between epigenetic modification and social/environmental cancer risks. This illustrates promising opportunities to leverage epigenetic data to inform future diagnostic and therapeutic targets. Although such strategies address biological mediators, they do not resolve upstream social determinants of preventable cancer risk (16). This will require a complementary approach that holistically addresses both biological and sociological root causes of cancer disparities.

## Translating social genomic frameworks into actionable cancer care strategies

**Figure 1:**
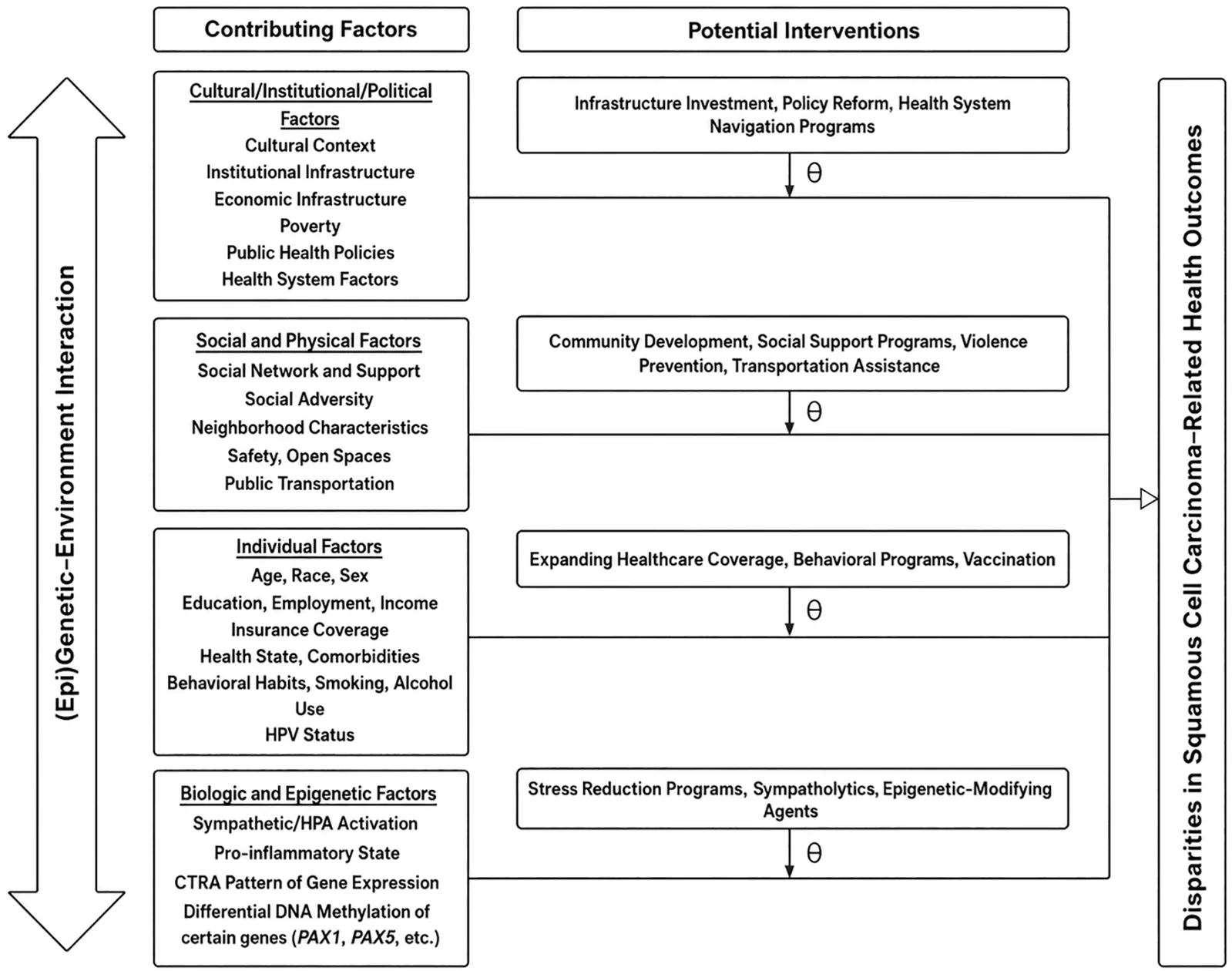
A proposed multilevel framework for translational epidemiology, adapted from Goel, Hernandez and Cole, (25), depicts the interconnectedness of SDOH at different levels influencing SCCa-related health outcomes. The model highlights pathways leading to outcome disparities and identifies points for potential intervention. SDOH, social determinants of health; SCCa, squamous cell carcinoma.

Goel, Hernandez, and Cole (25) have recently proposed a model to describe how multi-level societal, institutional, environmental, and community/individual SDoH can interface with genetic and physiologic inputs to yield cancer disparities. The authors have leveraged this model to conceptualize findings from their epidemiological studies correlating neighborhood disadvantage with breast cancer outcomes in their South Florida catchment area. Controlling for individual-level, tumor, and guideline-concordant treatment, they determined that living in disadvantaged conditions was an independent predictor of shorter survival (14). This model provides a strong foundation for uncovering broader risk interactions to guide future treatment strategies for SCCa. **Figure 1** (adapted from Goel et al.) illustrates a multilevel framework linking SDoH to SCCa-related outcomes disparities through interactions between environmental exposures, individual characteristics, and biological factors. The framework begins with broader cultural and institutional factors (e.g., poverty, public health policies, and health system infrastructure), which shape social and physical environments (e.g., neighborhood characteristics, safety, and transportation). These broader community-level factors, in turn, interact with individual-level characteristics such as income, insurance coverage, and behavioral habits. At the biological level, the model highlights neuroendocrine stress response, pro-inflammatory signaling, and epigenetic regulation of gene expression, that may mediate the effects of adverse social exposures on SCCa-related outcomes. **Figure 1** also identifies corresponding intervention points at each level, ranging from infrastructure investment and policy reform to community-based support programs, healthcare navigation, behavioral interventions, and emerging biologically targeted or epigenetic-modifying agents. By mapping these multilevel influences and intervention opportunities onto SCCa-related outcomes, this model provides a framework through which SDoH and biologic data can be integrated to identify vulnerable populations and guide targeted cancer care strategies.

It also is a crucial opportunity to intentionally seek direct cooperation among translational scientists, clinical trialists, cancer care providers, and patient community stakeholders to propel acceptance and dissemination. It is well known that socially vulnerable populations suffer from trust deficits with their care; their perspectives and responses to healthcare delivery systems can catalyze or hinder implementation (133, 134).

Translating this multi-level framework into actionable strategies represents a key barrier to informing personalized care and reducing cancer outcome disparities. Goel et al. (135) have suggested “geospatial genomics” as an approach to link geospatial models to multi-omics data to guide a better understanding of how environmental factors influence cancer biology. This approach enables the simultaneous integration of multi-omics data (epigenome, transcriptome, and patient exposome) with patient outcomes and characterizes interactions between tumor physiology and social context. Among candidate SDoH, home location of the patient residence is associated with outcomes at the level of census tract or zip code (136) and has been suggested as a composite measure of complex social barriers to care. Thus, geospatial analysis can provide a nuanced untangling of how related SDoH factors interact across specific environmental and social contexts (137). Area deprivation indices, such as the Area Deprivation Index (ADI) and the Social Vulnerability Index (SVI), are increasingly being captured in institutional electronic health records (EHRs) and hold promise for flagging patients for targeted psychosocial support alongside standard oncologic treatment. Though, systematic integration into real-time clinical workflows remains limited. Similarly, geospatial tools that incorporate genomic and neighborhood-level risk data could be integrated into cancer registry infrastructure or EHRs to identify high-risk communities, guide proactive screening, and support more equitable resource allocation.

Based on Diderichsen’s model (138), disparities in health outcomes originate from two major sources. The first encompasses potential hazard exposures and heightened vulnerability of certain populations to these exposures. The other involves resource constraints faced by these target populations when adverse outcomes do occur. Since neighborhood location is a key determinant of individual-level environment, social vulnerability, and physical access to healthcare facilities, geospatial analysis provides an opportunity to study, conceptualize, and address health disparities. This approach is particularly relevant to SCCa since incidence and mortality risks for this cancer type are highly influenced by environmental determinants and socioeconomic factors. Environmental risk factors of SCCa are heterogeneously distributed across regional locations. By mapping physical distribution of exposure insults and SCCa incidence, it is possible to triage communities that are disproportionately impacted by adverse exposures to relevant public and patient-level health interventions. For example, one study examined DNAm levels of candidate genes in HNSCCa patients and showed differential DNAm levels (and thus potential therapeutic targets) across zip codes which are associated with survival outcomes (67). Geospatial analysis holds particularly high potential to assess social resource deficits and healthcare access improvement opportunities for vulnerable cancer patient populations. Our group has previously used this approach to characterize disparities in an important cancer care quality outcome, specifically unplanned interruption rates in HNSCCa radiation treatment in Southwestern Tennessee (139). We showed that patient home location in zip codes with majority-minority populations was associated with a higher risk for treatment interruption. Our group is now prospectively investigating the feasibility and clinical impact of community-based cancer navigation directed at patients from these at-risk neighborhoods.

Therapeutic measures targeting stress response pathways (such as beta blocker or epigenetic modifiers) suggest that future clinical trials may benefit from integrating SDoH data, including neighborhood deprivation and biological measures of chronic stress, to determine whether socially disadvantaged patients derive differential benefit from these agents. It is worth noting that the biological mechanisms described here do not operate independently of their social context and cannot be fully addressed through molecular intervention alone. The epigenetic embedding of neighborhood adversity in SCCa represents the biological imprint of structural inequity. Therefore, meaningfully reducing cancer outcome disparities requires a dual approach: using the biological embedding of adversity to stratify risk, refine prognostic biomarkers, and develop targeted therapies, while simultaneously pursuing the structural and policy-level interventions outlined in **Figure 1**.

## Conclusion

We are only beginning to understand the complex relationships linking social risks, cancer biology, and health outcomes. Future work will need to identify genetic targets, epigenetic events, and pathophysiologic pathways by which adverse social exposures impact specific patient populations. Such discoveries promise to guide the intelligent selection of personalized preventive and therapeutic interventions to address each patient and community’s unique vulnerabilities, ensuring equitable and effective treatment for all cancer populations. Future social genomics studies could integrate geospatial analysis with epigenomic data to capture a data continuum spanning socioecological factors, genetic and epigenetic markers, and downstream SCCa-related health outcomes. This comprehensive approach promises to enhance our understanding of intertwined mechanisms driving SCCa pathogenesis, accurately identify specific vulnerable populations, and ultimately guide the development and evaluation of targeted interventions (140) at social, individual, and biological levels.
