# Correction: Social genomics and cancer – untangling nature from nurture in squamous cell carcinoma outcomes

**DOI:** 10.3389/fonc.2026.1951762

**Published:** 2026-08-11

**Authors:** Parnian Kheirkhah Rahimabad, Arash Shaban-Nejad, Robert Davis, D. Neil Hayes, Liza Makowski, David L. Schwartz

**Affiliations:** 1 Departments of Radiation Oncology, College of Medicine, University of Tennessee Health Science Center, Memphis, TN, United States; 2 Department of Pediatrics, Center for Biomedical Informatics, College of Medicine, University of Tennessee Health Science Center, Memphis, TN, United States; 3 Division of Hematology-Oncology, Department of Medicine, College of Medicine, University of Tennessee Health Science Center, Memphis, TN, United States; 4 Departments of Preventive Medicine, College of Medicine, University of Tennessee Health Science Center, Memphis, TN, United States

**Keywords:** cancer outcomes, epigenetics, social determinants of health, social genomics, squamous cell carcinoma

There was a mistake in **Figure 1** as published. There are 2 typos in the figure. The corrected **Figure 1** appears below.

**Figure 1:**
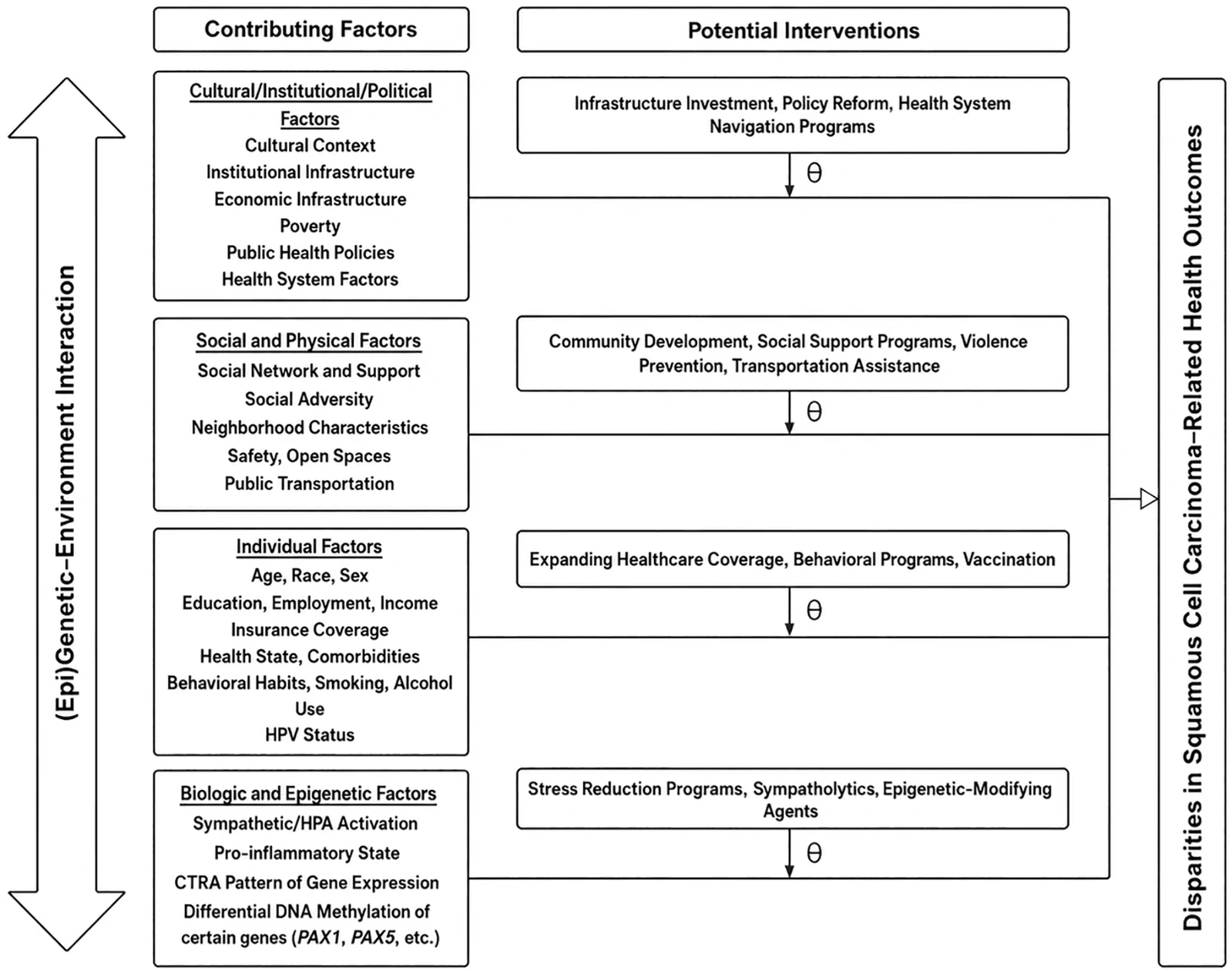
A proposed multilevel framework for translational epidemiology, adapted from Goel et al. (Goel, Hernandez and Cole, 2024), depicts the interconnectedness of SDoH at different levels influencing SCCa-related health outcomes. The model highlights pathways leading to outcome disparities and identifies points for potential intervention. SDoH, social determinants of health; SCCa, squamous cell carcinoma.

