# Supplementary material for: Social genomics and cancer – untangling nature from nurture in squamous cell carcinoma outcomes
Source: Front Oncol. 2026 Jul 20;16:1661519. doi: 10.3389/fonc.2026.1661519 (PMC13429487; doi:10.3389/fonc.2026.1661519)
Supplement: Supplementary file 1 [file Table1.docx]

Supplementary Table 1. Select studies on epigenetic targeting agents in treating Squamous cell carcinomas (SCCa).

| **Mechanism of Action** | **Author, year** | **SCCa type** | **Study design and population** | **Key findings** |
| --- | --- | --- | --- | --- |
| **Beta adrenergic blockade** | Lucido et al., 2018 | HPV+ HNSCCa | In vitro (murine and human cell lines), in vivo (murine model) | Propranolol treatment significantly decreased tumor cell proliferation (in vitro, murine and human cell lines). It also suppressed tumor growth and metastasis compared with controls in murine models (in vivo). Propranolol showed additive antitumor effects when combined with standard chemoradiation, resulting in reduced tumor burden and delayed progression. |
|  | Shibuya et al., 2022 | OSCCa | In vitro (human cell line) | Propranolol reduced tumor cell viability and sensitized OSCCa cells to cisplatin and 5-fluorouracil, demonstrating significantly lower cell survival compared with chemotherapy alone. |
|  | Kim et al., 2017 | HNSCCa | Clinical (retrospective cohort) | In HNSCCa patients, beta blocker use was associated with worse overall survival (HR 1.54, 95% CI 1.17-2.05), a trend toward worse cancer-specific survival (HR 1.49, 95% CI 0.99-2.22), and significantly worse non-cancer survival (HR 1.80, 95% CI 1.17-2.79). |
|  | Chen et al., 2023 | HNSCCa | Clinical (retrospective cohort) | In HNSCCa patients, beta blocker use was associated with worse overall survival (HR, 1.67; 95% CI, 1.06-2.62) and worse disease-free survival (HR 1.67; 95% CI, 1.06-2.63). |
| **DNA Methyltransferase (DNMT) Inhibition** | Biktasova et al., 2017 | HPV+ HNSCCa | In vitro (human cell lines and primary tumor culture), in vivo (murine model) | 5-azacytidine treatment significantly inhibited tumor growth and induced cell death in HPV+ HNSCCa models. 5-azacytidine decreased expression of matrix metalloproteinases and activated interferon response in HPV+ HNSCCa cells. In murine model, 5-azacytidine inhibited tumor blood vessel invasion. |
|  | Viet et al., 2014 | HNSCCa (tongue SCCa) | In vitro (human cell lines), in vivo (murine model) | Decitabine restored cisplatin sensitivity in cisplatin-resistant cells. Combination therapy resulted in sustained tumor growth inhibition in vivo. |
|  | Mahesh et al., 2010 | LSCCa | In vitro (human cell lines) | 5-azacytidine reduced growth in LSCCa cells in a dose-dependent manner and reversed hypermethylation of a tumor suppressor (RASSF1a) promoter. |
|  | Burtness et al. / ongoing trial | HPV+ HNSCCa | Phase II randomized clinical trial in adults with resectable HPV+ oropharynx SCCa | This ongoing trial is to compare pre-operative 5-azacytidine, nivolumab, or the combination in HPV+ oropharyngeal SCCa patients in terms of immune-related pathologic response, tumor immune infiltration, and safety. The study aims to determine whether DNMT inhibition enhances sensitivity and anti-tumor immune response to PD-1 blockade. |
| **Histone Deacetylase (HDAC) Inhibition** | Teknos et al., 2019 | HNSCCa | Phase clinical trial in adults with advanced HNSCCa, single arm study | Combination of vorinostat, cisplatin and radiation therapy was well tolerated and demonstrated tumoricidal activity. |
|  | Chen et al., 2014 | Recurrent/metastatic HNSCCa | Phase II clinical trial | Combimation of vorinostat capecitabine in recurrent and/or metastatic HNSCCa showed limited clinical activity. Of 16 patients, 2 had partial response. Median PFS was 2.3 months (95% CI 1.2-3.6) and median OS 10.8 months (95% CI 5.0-not reached). Regimen showed limited clinical activity |
|  | Ma et al., 2015 | ESCCa | In vitro (human cell lines) | Trichostatin A treatment suppressed proliferation in a dose-dependent manner, reducing cell viability, induced G1/G0 arrest and significantly increased apoptosis. |
|  | Hoshino et al., 2005 | ESCCa | In vitro (human cell lines), in vivo (murine models) | FK228 showed potent antiproliferative activity by inducing cell arrest and apoptosis. FK228 increased expression of *Prdx1* gene through acetylating histones H3 and H4 of its promoter.  In vivo, FK228 showed significant tumor regression. |
| **BET (Bromodomain and Extra-Terminal) Inhibition** | Zhang et al., 2022 | HNSCCa | In vivo (murine models) | BET inhibition with JQ1 significantly boosted antitumor immunity through different mechanisms including increasing CD8⁺ tumor-infiltrating lymphocytes. BET inhibition increased MHC-I expression in HNSCCa. Combination therapy (JQ1 + anti-PD-1) produced synergistic tumor suppression. |
|  | Yang et al., 2022 | ESCCa | In vitro (human cell lines) | BRD4 inhibition (JQ1) suppressed cell proliferation but significantly increased cell migration by epithelial-mesenchymal transition induction (decreased E-cadherin, increased vimentin) and autophagy activation. Autophagy inhibition reversed the JQ1-induced migration. |

SCCa: squamous cell carcinoma; HNSCCa: head and neck SCCa; OSCCa: oral SCCa; LSCCa: Lung SCCa; ESCCa: Esophageal SCCa
